# Homocysteine levels in first-episode patients with psychiatric disorders

**DOI:** 10.3389/fpsyt.2024.1380900

**Published:** 2024-05-23

**Authors:** Ning Fan, Wenxuan Zhao, Yajun Yun, Luyuan Bai, Huimei An, Qi Zhang, Jiangling Yan, Fengmei Fan, Xiaole Han, Fude Yang

**Affiliations:** ^1^ Beijing Huilongguan Hospital, Peking University Huilongguan Clinical Medical School, Beijing, China; ^2^ Wuxi Mental Health Center, Wuxi, China

**Keywords:** homocysteine, depression, bipolar disorder, schizophrenia, first-episode

## Abstract

A high homocysteine (Hcy) level is a risk factor for schizophrenia, depression, and bipolar disorder. However, the role of hyperhomocysteinemia as either an independent factor or an auxiliary contributor to specific psychiatric symptoms or disorders remains unclear. This study aimed to examine Hcy levels in first-episode inpatients with psychotic symptoms and various psychiatric diseases to elucidate the association between Hcy levels and psychiatric disorders. This study enrolled 191 patients (aged 18–40 years) with psychiatric disorders. Seventy-five patients were diagnosed with schizophrenia, 48 with acute and transient psychotic disorders, 36 with manic episodes with psychosis, 32 with major depressive episodes with psychosis, and 56 healthy controls. Serum Hcy levels were measured using the enzyme cycle method. A Hcy concentration level of > 15 μmol/L was defined as hyperhomocysteinemia. Hcy levels were significantly higher in first-episode patients with psychiatric disorders compared to healthy controls (5.99 ± 3.60 vs. 19.78 ± 16.61 vs. 15.50 ± 9.08 vs. 20.00 ± 11.33 vs. 16.22 ± 12.06, *F* = 12.778, *P* < 0.001). Hcy levels were significantly higher in males with schizophrenia, acute and transient psychotic disorder, and major depressive disorder but not in mania [schizophrenia, (*t* = -4.727, *P* < 0.001); acute and transient psychotic disorders, (*t* = -3.389, *P* = 0.001); major depressive episode with psychosis, (*t* = -3.796, *P* < 0.001); manic episodes with psychosis, (*t* = -1.684, *P* = 0.101)]. However, serum Hcy levels were not significantly different among the psychiatric disorder groups (*F* = 0.139, *P* = 0.968). Multivariate linear regression showed that males had an increased risk for homocysteinemia. (95% CI = 8.192–15.370, *P* < 0.001). These results suggest that first-episode patients with psychiatric disorders have higher Hcy levels than in the general population, and men are at greater risk for psychiatric disorders. In conclusion, elevated Hcy levels may contribute to the pathogenesis of first-episode patients with psychotic symptoms.

## 1 Introduction

Homocysteine (Hcy) is an intermediate product of the methionine cycle that provides methyl groups for methylation reactions involved in the one-carbon cycle (1). Homocysteine metabolism occurs through methylation or transsulfuration pathways, which are influenced by methylene-tetrahydrofolate reductase (MTHFR) (2). Folic acid, vitamin B6, and vitamin B12 are methyl providers or coenzymes in these pathways (3).

Elevated Hcy levels of > 15 mmol/L are defined as hyperhomocysteinemia (Hhcy) (4). Both elevated Hcy and Hhcy levels are associated with several neurological and psychiatric disorders, such as stroke (5), major cognitive disorders (6), schizophrenia (SZ) (7), bipolar disorder (BD) (4), and major depression (8). These factors contribute to the pathogenesis of these disorders through multiple pathways (3). Elevated maternal Hcy levels could reduce the oxygen supply to the fetus and affect the development of fetal brain structures, thereby increasing the risk of SZ. Furthermore, modulated levels of neurotransmitters such as dopamine, norepinephrine, epinephrine, serotonin, and melatonin suggest an association between Hcy levels and major depression (1, 9). Hcy may also be related to depression through nonvascular brain damage resulting from neurotoxicity or methylation pathway dysfunction (10). Furthermore, Hcy exacerbates oxidative stress responses, increases neural vulnerability, and limits neuronal recovery from stress injuries in patients with BD (11, 12).

In most studies on SZ, BD, and depression, higher Hcy or Hhcy levels were observed in the disease group than in the control group (7, 13). However, whether Hhcy is an independent or add-on risk factor for specific psychiatric symptoms or disorders is unclear. Examples of psychiatric symptoms include depression, positive/negative psychotic symptoms (e.g., hallucinations, delusions, apathy, abulia). A meta-analysis was conducted to validate previous research conclusions comprehensively and further clarify the relationship between Hcy and psychiatric disorders (13). In a large sample study, Hcy levels of patients with SZ were higher than those of healthy controls (HCs), regardless of sex (14). However, age was inversely proportional to Hcy levels only in male patients. *MTHFR 677 C>T* increases the risk of SZ, and a large difference in the ethnicity of humans was observed (14). Moradi et al. (13) included 46 studies in a quantitative analysis, the Hcy level was higher in patients with depression compared to HCs. Salagre et al. (15) included nine studies in a meta-analysis, and elevated Hcy levels were observed in patients with BD. However, all patients were receiving psychopharmacological treatment (15).

Previous studies have mostly compared Hcy levels in SZ, BD, or depression separately (8, 11, 12, 16). Few studies have simultaneously compared the Hcy levels between two of these diseases, and the homogeneity of patients was not considered. Therefore, simultaneously investigating multiple diseases is crucial, and symptoms and disease status should be considered. In addition, the confounding effects of age, medication, race, and chronic illness also need to be controlled because these biological factors could influence Hcy levels (8, 16). For example, Hcy levels decreased after antipsychotic therapy, and the severity of psychotic symptoms was associated with higher Hcy levels (17). Hcy is also associated with age (8). However, while different diagnostic tools have a crucial impact on the study results, whether confounding factors were controlled could also have significantly impacted the results of these studies (13). Therefore, the cohort in this study comprised first-episode, younger individuals (18–40 years) during the relatively early stages of psychiatric illnesses (illness duration < 5 years). All patients had psychotic symptoms. Thus, the confounding effects of age, medication, and illness chronicity were minimized.

To further validate a hypothesis, psychiatric disorders may share a common or similar pathophysiological basis. This study aimed to investigate: 1) whether serum Hcy levels are altered in patients with first-episode psychiatric disorders, 2) whether serum Hcy levels show intergroup differences among patients with SZ, acute and transient psychotic disorders (ATPD), manic episodes with psychosis (ME), and major depressive episode with psychosis (MD), and 3) whether higher Hcy levels are related to disease type, which may provide a biological indicator for the diagnosis and differential diagnosis of different psychiatric disorders.

## 2 Materials and methods

### 2.1 Participants

**Figure 1:**
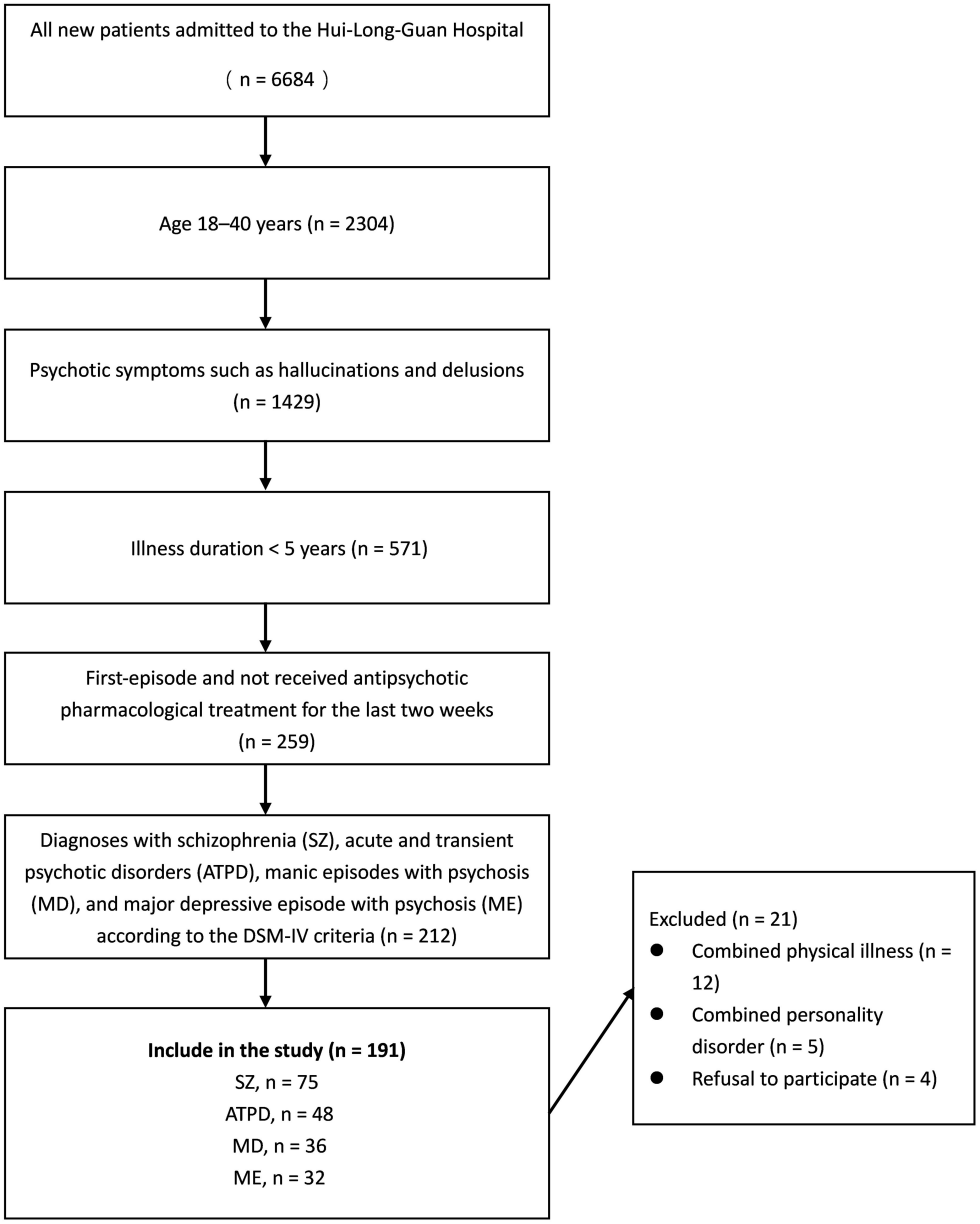
Flow chart of the patient selection process.

This study was conducted at a single site, and participants were recruited from hospitalized patients at Hui-Long-Guan Hospital, a city-run psychiatric hospital in Beijing, China. All participants were first-episode and had not received antipsychotic pharmacological treatment for the two weeks preceding the initiation of the study. All patients had an illness duration of < 5 years and psychotic symptoms such as hallucinations and delusions. The specific disorder was diagnosed according to the Diagnostic and Statistical Manual of Mental Disorders, Fifth Edition (DSM-5) diagnostic criteria by two experienced psychiatrists with more than 5 years of experience. The exclusion criteria were as follows: 1) combined severe organic disease; 2) use of drug and vitamin supplements that can influence serum Hcy levels; 3) negative family history of psychiatric disorders; and 4) pregnant and lactating women. Seventy-five patients with SZ, 48 with ATPD, 32 with MD, and 36 with ME were included (**Table 1** and **Figure 1**). Fifty-six healthy control participants were selected from the local communities that had no severe mental illnesses according to the DSM-5 criteria. The exclusion criteria for the HCs were the same as those for the patient groups.

**Table 1:** Demographic information of patients with psychiatric disorders and healthy controls.

	HC (n = 56)	SZ (n = 75)	ATPD (n = 48)	ME (n = 32)	MD (n = 36)	*F*/*χ* ^2^	*P*
Age (y)	27.36 (7.78)	27.20 (7.14)	25.21 (5.66)	27.33 (7.76)	24.25 (5.80)	1.775	0.134
Female	37 (66%)	35 (47%)	28 (58%)	15 (47%)	22 (61%)	7.121	0.130
Male	19 (34%)	40 (53%)	20 (42%)	21 (53%)	10 (39%)		
BMI (kg/m^2^)	21.82 (5.08)	21.91 (3.04)	22.04 (3.07)	22.33 (4.25)	21.00 (1.90)	0.929	0.448
Hcy (µmol/L)	5.99 (3.60)	19.78 (16.61)	15.50 (9.08)	20.00 (11.33)	16.22 (12.06)	12.778	< 0.001
Female	5.38 (3.27)	11.24 (6.26)	12.11 (7.63)	16.34 (5.74)	11.68 (5.94)	11.496	< 0.001
Male	7.17 (4.01)	27.25 (14.15)	20.25 (8.96)	22.63 (10.58)	26.2 (11.06)	5.850	< 0.001
Hhcy (%)	0 (0%)	27 (36%)	22 (46%)	24 (67%)	12 (38%)	10.026	0.018

Data are summarized as the mean (SD) and frequency (%). No significant difference was observed between patients and HCs regarding age, BMI, and sex using analysis of variance and chi-square test. Compared to the HC group, all patient groups had higher Hcy levels; however, no significant differences in Hcy levels were observed among patients with SZ, ATPD, ME, or MD (all *P* > 0.05). The prevalence rates of Hhcy in patient groups with SZ, ATPD, ME, and MD were significant (*χ*2 = 10.026, *P* = 0.018). SZ, schizophrenia; ATPD, acute and transient psychotic disorders; ME, manic episodes with psychosis; MD, major depressive episode with psychosis; HC, healthy controls; BMI, body mass index; Hcy, homocysteine; Hhcy, hyperhomocysteinemia.

The Ethics Committee of Beijing Hui Long Guan Hospital approved all experimental protocols. Informed consent was obtained from all patients. All methods were performed in accordance with the Declaration of Helsinki.

### 2.2 Hcy measurement

Venous blood (4 mL) was collected from each patient between 06:00 and 07:00 AM after 12 h of fasting after the patient’s diagnosis was confirmed. The serum samples were centrifuged at 3000 rpm for 5 min and immediately stored at −80°C. Sandwich enzyme-linked immunosorbent assay kit (catalog no: EIA-2925, DRG Instruments GmbH, Marburg, Germany) was used to measure the Hcy levels of the participants. Hcy concentrations of > 15 μmol/L were defined as Hhcy (8).

### 2.3 Statistical analyses

The results are presented as the mean ± standard deviation (SD) to describe quantitative variables. A one-way analysis of variance (ANOVA) test was used to compare the demographics and mean Hcy levels in the five groups (SZ, ATPD, ME, MD, and HCs). Levene’s test for homogeneity was significant (*P* < 0.05). Tamhane’s T2 test was used to make multiple comparisons. Differences in serum Hcy levels between male and female participants were analyzed using an independent-sample *t*-test. The prevalence of Hhcy was analyzed using the *χ* ^2^ test. Multivariate linear regression models were used to (age, sex, body mass index [BMI], and diagnosis) associated with Hcy concentrations in patients with psychiatric disorders. Psychiatric disorders such as SZ, ATPD, MD, and ME were defined as diagnostic variables in this analysis. Generalized linear models were used to validate the results, considering the influence of confounding factors such as age and sex. IBM SPSS Statistics for Windows (version 27.0) was used for data analysis (18). *P* < 0.05 was considered statistically significant.

## 3 Results

### 3.1 Demographic data

One hundred ninety-one participants with psychiatric disorders were included. Fifty-six healthy participants were included as HCs. No significant differences in age, sex, or BMI were observed between the patient groups and HCs (all *P* > 0.05). *Post-hoc* comparison was also performed using Tamhane’s T2 test; no differences in general demographic information (such as age and BMI) were observed among the different patient groups (all *P* > 0.05). No significant sex differences were observed among the patient groups using the chi-square test (*P > 0.05*) (**Table 1**).

### 3.2 Difference in homocysteinemia levels between patients and controls

**Figure 2:**
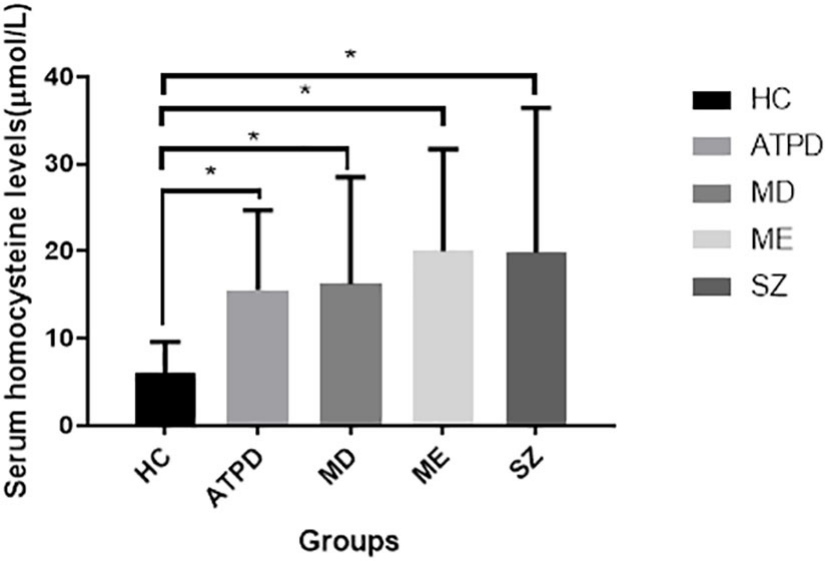
Serum homocysteine levels in different groups. All patient groups had higher Hcy levels (*P* < 0.01) than in the HC group. However, no significant difference in Hcy levels was observed among patients with these disorders (all *P* > 0.05). *P < 0.01 compared to the healthy control group. Hcy, homocysteine; HC, healthy controls; SZ, schizophrenia; ATPD, acute and transient psychotic disorders; MD, major depressive episode with psychosis; ME, manic episodes with psychosis.

Compared to the HC group, all patient groups had higher Hcy levels (5.99 ± 3.60 vs. 19.78 ± 16.61 vs. 15.50 ± 9.08 vs. 20.00 ± 11.33 vs. 16.22 ± 12.06, *F* = 12.778, *P* < 0.001); however, no significant differences in Hcy levels were observed among patients with SZ, ATPD, ME, or MD (all *P* > 0.05) (**Table 1** and **Figure 2**). Then, adjusting for age and sex, significant differences in Hcy levels were still observed between patients and HCs (*F* = 18.265, *P* < 0.001) but not in different patient groups (*F* = 0.139, *P* = 0.968).

The healthy group was excluded to further observe the incidence of Hhcy in the disease group and to assess the differences in Hhcy rates in patients in different disease groups alone. Prevalence rates of Hhcy in these patient groups with psychosis were significant [SZ, 27/75 (36.00%) vs. ATPD, 22/48 (45.83%) vs. ME, 24/32 (66.67%) vs. MD, 12/36 (37.50%), *χ* ^2^ = 10.026, *P* = 0.018] (**Table 1**).

### 3.3 Effect of sex on serum Hcy levels

**Figure 3:**
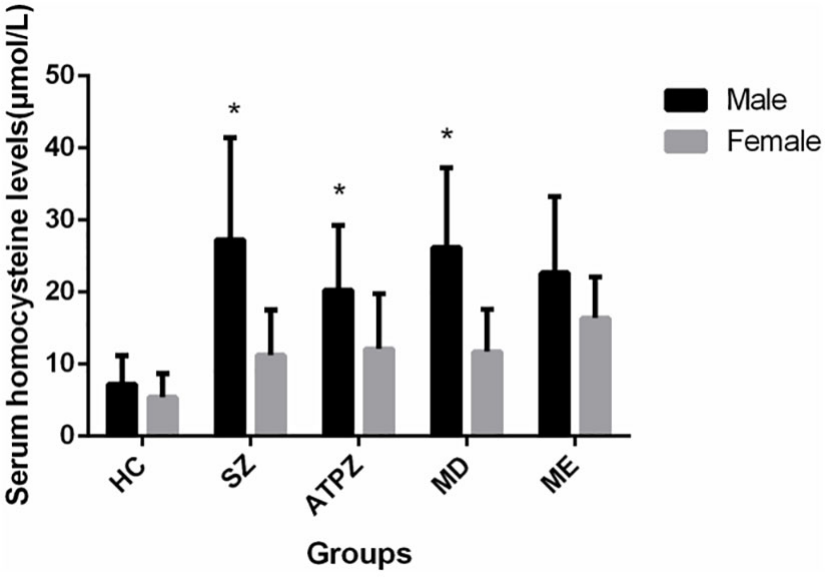
Sex differences in Hcy levels in patients and healthy controls. Male patients with SZ, APTD, and MD had significantly higher Hcy levels than female patients (*P* < 0.01). *P < 0.01 compared to the female group.

Significant sex differences were observed in serum Hcy levels in three patient groups. Male patients had significantly higher Hcy levels than female patients [SZ, (*t* = -4.727, *P* < 0.001); ATPD, (*t* = -3.389, *P* = 0.001); MD, (*t* = -3.796, *P* < 0.001)] except in patients with ME (*t* = -1.684, *P* = 0.101) (**Figure 3**). Moreover, most participants with Hhcy were men [SZ, 23/27 (*χ* ^2^ = 17.197, *P* < 0.001); ATPD,14/22 (*χ* ^2^ = 16.121, *P* < 0.001); ME, 15/24 (*χ* ^2^ = 0.514, *P* = 0.473); MD, 8/12 (*χ* ^2^ = 11.210, *P* < 0.001)].

Two separate ANOVAs were conducted for males and females. Significant differences in Hcy levels were observed in males and females between patients and HCs (males: 7.17 ± 4.01 vs. 27.251 ± 4.15 vs. 20.25 ± 8.96 vs. 22.63 ± 10.58 vs. 26.21 ± 11.06, *F* = 5.850, *P* < 0.001; females: 5.38 ± 3.27 vs. 11.24 ± 6.36 vs. 12.11 ± 7.63 vs. 16.34 ± 5.74 vs. 11.68 ± 5.94, *F* = 11.496, *P* < 0.001). However, no significant differences in Hcy levels were observed among patient groups (female: *F* = 2.146, *P* = 0.100; male: *F* = 0.671, *P* = 0.572) (**Table 1**).

### 3.4 Risk factors for Hcy levels

A multivariate linear regression model was used to evaluate factors associated with Hcy levels, including age, sex, BMI, and diagnosis (including SZ, ATPD, ME, or MD). Male sex and psychiatric disorders are factors associated with Hcy (**Table 2**).

**Table 2:** Multivariate linear regression model for homocysteine concentrations in patients with psychiatric disorders.

	B	β coefficient	95% CI	*P* value
Sex	11.781	0.440	8.192–15.370	<0.001
Age	0.027	-0.013	-0.289 – 0.236	0.842
BMI	0.232	0.055	-0.334 – 0.799	0.419
Diagnosis	-0.190	-0.239	-1.756–1.376	0.811

CI, confidence interval; BMI, body mass index; SZ, schizophrenia; ATPD, acute and transient psychotic disorders; ME, manic episodes with psychosis; MD, major depressive episode with psychosis.

## 4 Discussion

To the best of our knowledge, this study is the first to investigate Hcy levels in inpatients with SZ, ATPD, ME, and MD in a Chinese population. In contrast to previous studies that examined Hcy levels in individual or two disorders, this study included patients with first-episode, similar clinical symptoms but with different disease diagnoses to better determine whether Hcy elevation is specific to different psychiatric disorders. In the present study, patients with SZ, ATPD, ME, and MD had higher serum Hcy levels than HCs; however, no differences in Hcy levels were observed between intergroup psychiatric disorders. In agreement with previous studies (8, 12, 16, 19), Hcy concentrations were elevated in Han Chinese with SZ (16, 19), and/or first-episode SZ (17). Similarly, elevated Hcy levels were observed in participants with ME and MD (9, 11, 12).

The cause of elevated serum Hcy levels in patients with psychiatric disorders is unclear; however, folic acid, vitamin B12 and B6 deficiencies, and *MTHFR* genetic polymorphism alterations may lead to increased Hcy levels (2, 3). A positive association between lower vitamin B12 levels and Hcy dysregulation was subsequently observed in patients with depression (10). A defect in the methylation processes owing to vitamins B6, B12, or folate deficiencies is central to neuropsychiatric manifestations (3). Hcy is remethylated to methionine via the 5-methyltetrahydrofolate pathway, with vitamin B12 acting as a cofactor and folic acid providing the methyl group transferred in the reaction (2). Alternatively, Hcy can be condensed with serine to form cystathionine in a reaction where VB6 acts as a coenzyme. Folic acid and B vitamins are reduced in patients with SZ and affective psychosis (3, 20). The folate cycle is the major pathway for re-methylating and reducing Hcy levels. The other re-methylation pathway requires the participation of betaine, which is transformed from choline, an essential substance competed for by bacterial microbiota (21). A correlation has been reported between gut microbiota and psychiatric disorders (22). Therefore, microbiota can either promote Hcy production or pose as a risk factor for psychiatric disorders. Further studies integrating different omics are required. Multi-omics studies will potentially generate valuable insights into the pathophysiology of psychiatric diseases and identify their biomarkers.

Furthermore, changes in gene polymorphisms are essential for Hhcy development in patients with psychiatric disorders. An association exists between changes in *MTHFR* gene polymorphisms and elevated Hcy levels, with two common polymorphisms of the *MTHFR* gene mutations being C677T and A1298C (2). These two polymorphisms may lower MTHFR activity and increase Hcy levels (2). Elevated Hcy levels and *MTHFR* gene polymorphisms are associated with increased risks of SZ, ME, and MD (23).

Elevated Hcy levels can also be attributed to advanced age (19). Age is a crucial risk factor for high Hcy levels, and a positive correlation has been observed between age and Hcy levels in the general population. Hcy levels are also correlated with age among patients with depression (9). In addition, older patients with SZ have a relatively high risk of Hhcy (16). The increase in Hcy concentration with age may be attributed to differences in dietary habits and lifestyles, as well as the impact of healthy development. Dietary factors influence Hcy, and variations in lifestyle and dietary habits can lead to differences in nutrient intake, thereby affecting Hcy levels. Moreover, healthy development necessitates a high proportion of creatine synthesis. This metabolic reaction is the primary consumer of methyl donated by S-adenosylmethionine, which is formed during the conversion of Hcy to Hhcy (24). However, the current study did not identify a correlation between age and Hcy levels, which may be related to the narrow age range of our participants. Additionally, all the research participants were from the same ethnic group, and those in the same age group mostly had similar dietary and lifestyle habits, possibly contributing to this result. Therefore, our research conclusions cannot yet be generalized to the general population. Future studies including patients from a broader age range with varying dietary and lifestyle habits are required to further validate the relationship between Hcy and age.

In addition, male patients have significantly higher Hcy and Hhcy levels than those of female patients (6, 11, 12), consistent with the findings of this study. However, the reasons for the preponderance of male patients remain unclear. Higher Hcy levels in male patients may be related to hormone levels, metabolic characteristics, and genetic polymorphisms (25). In contrast, Hcy levels were decreased in pregnant women or pre- and post-menopausal women who received hormone replacement therapy (26). Moreover, estrogen substantially influences Hcy biosynthesis. A relationship between Hcy levels and BMI also exists (4). Hcy levels are higher in individuals with obesity, and high Hcy levels promote the development of abnormal lipid metabolism. Male patients are more likely to develop metabolic syndrome (25). The effect of MTHFR gene polymorphisms on Hcy also showed considerable sex differences.

Considering the potential effects of age and sex on Hcy, this study used age and sex as covariates and covariance analysis to control these confounding factors. The results were stable and indicated that homocysteine levels do not differ according to diagnosis. Similar to the results in our study, another study that included 100 patients with SZ or BD did not observe a difference in Hcy levels between the two disorders (8). Notably, Hcy levels influence the symptoms of psychiatric disorders. For example, increased Hcy levels were associated with an increase in the severity of negative symptoms in schizophrenia, which reduced after treatment (17). Furthermore, a significant association was observed between depression symptoms and Hcy levels (27). However, Hcy levels do not affect mania symptoms (11). These results suggest that the psychopathophysiologies of these disorders may share common neural substrates. Based on various observations, oxidative stress may be associated with the pathophysiological mechanisms of mental disorders. Simultaneously, Hcy may act as an oxidant in various model systems. Most serum Hcy is protein-bound, and only a small proportion is an unbound portion of Hcy (28), and the majority of the free reduced Hcy is oxidized to a dimer. Alternatively, Hcy can be combined with cysteine to form a mixed disulfide. These oxidized Hcy forms are potentially neurotoxic, leading to risks of glutamate toxicity, increased intracellular levels of ionized calcium, reactive oxygen species (ROS), apoptotic transformation, and neurodegenerative processes (1, 8, 12). In addition, increased Hcy levels with simultaneously reduced folate levels could also increase oxidative stress. Folate has been implicated in direct ROS scavenging and is an antioxidant *in vivo*. Oxidative stress degrades folate. An increase in Hcy levels exacerbates oxidative stress and mitochondrial dysfunction, endangering dopaminergic neurons and thereby increasing the risk of depression (28). Oxidative stress and Hcy-regulated pathways are potential factors that hamper functional brain connectivity in BD (29). A close correlation exists between high Hcy levels, oxidative stress, and pathophysiological processes of SZ (30).

Hcy is converted to methionine, this pathway ultimately provides a methyl group for methylation that is also used for DNA methylation and is known to have a substantial role in various psychiatric diseases (23). For example, genomic DNA methylation directly correlates with Hcy levels in patients with SZ (31). Furthermore, DNA methylation could be a promising epigenetic pathological biomarker for depression and potentially predict antidepressant treatment efficacy (32). Most studies suggest abnormalities in DNA methylation across various psychiatric disorders, similar to the findings of oxidative stress studies (30). However, whether differences in DNA methylation exist among different disorders remains unclear. SZ, BD, and MD could be different manifestations of the same genetic substrates (23). These conditions result from the interaction of multiple genetic and environmental factors, involving multiple genes with small interactive and additive effects. These results suggest that Hcy metabolism and psychiatric disorders are complex issues that must be investigated in the future.

This study has several limitations. First, the small sample size of this study may have impacted the findings, and future studies could be based on the preliminary Hcy measurement results in this study and performed detailed sample size estimates to obtain more accurate conclusions. Second, this study was a cross-sectional study that only identified an association between Hcy and psychiatric disease but did not determine causation. Third, only the Chinese population was enrolled in this study, limiting its generality with respect to ethnic groups. Fourth, while the multivariate linear regression model is robust for assessing factors associated with Hcy levels, determining the causal or correlative relationship between gender and Hcy levels requires dialectical analysis and judgment based on model fit and potential limitations.

## 5 Conclusion

Elevated Hcy levels were associated with various psychiatric disorders. However, no differences in Hcy levels were observed across individual psychiatric disorders. The investigation of Hcy in psychiatric diseases has crucial clinical implications. Identifying the role of Hcy in psychiatric diseases and assessing the differences in Hcy levels among various psychiatric diseases provides a theoretical basis for further understanding its involvement in their onset and treatment. This can lead to the development of novel therapeutic approaches or personalized treatment methods for psychiatric diseases. Considering the limitations of the current study, which could affect the generalizability of the findings, future studies should investigate the relationship between Hcy levels in various patient populations, including patients without psychiatric disorders encompassing a broader age range while controlling for cofounders (such as vitamin B12, vitamin B6, folate, and *MTHFR* genotypes). Additionally, the clinical symptoms of patients must be assessed to elucidate the relationship between Hcy, psychiatric disorders, and clinical symptoms. This research would provide further insight into this interesting association and determine the strength of Hcy as a biomarker.

## Data availability statement

The raw data supporting the conclusions of this article will be made available by the authors, without undue reservation.

## Ethics statement

The studies involving humans were approved by Ethics Committee of Beijing Hui Long Guan Hospital. The studies were conducted in accordance with the local legislation and institutional requirements. The participants provided their written informed consent to participate in this study.

## Author contributions

NF: Data curation, Writing – original draft. WZ: Investigation, Writing – original draft. YY: Conceptualization, Writing – original draft. LB: Project administration, Supervision, Writing – original draft. HA: Writing – review & editing, Visualization. QZ: Data curation, Formal analysis, Writing – review & editing. JY: Investigation, Project administration, Writing – original draft. FF: Validation, Writing – review & editing. XH: Project administration, Resources, Writing – review & editing. FY: Conceptualization, Funding acquisition, Project administration, Writing – review & editing.
